# How surface coatings on titanium implants affect keratinized tissue: A systematic review

**DOI:** 10.1002/jbm.b.35025

**Published:** 2022-02-01

**Authors:** Casper E. Van den Borre, Brandaan G. R. Zigterman, Maurice Y. Mommaerts, Annabel Braem

**Affiliations:** ^1^ Doctoral School of Life Sciences and Medicine Vrije Universiteit Brussel Brussels Belgium; ^2^ European Face Centre Universitair Ziekenhuis Brussel, Vrije Universiteit Brussel Brussels Belgium; ^3^ Department of Materials Engineering, Biomaterials and Tissue Engineering Research Group KU Leuven Leuven Belgium

**Keywords:** coating, implant, keratinized tissue, soft‐tissue integration, titanium, transcutaneous implant

## Abstract

Apart from osseointegration, the stability and long‐term survival of percutaneous titanium implants is also strongly dependent on a qualitative soft‐tissue integration in the transcutaneous region. A firm connective tissue seal is needed to minimize soft‐tissue dehiscence and epithelial downgrowth. It is well‐known that the implant surface plays a key role in controlling the biological response of the surrounding keratinized tissue and several coating systems have been suggested to enhance the soft‐tissue cell interactions. Although some promising results have been obtained in vitro, their clinical significance can be debated. Therefore, the purpose of this systematic review is to gain more insight into the effect of such coatings on the interface formed with keratinized soft‐tissue in vivo. A comprehensive search was undertaken in March 2021. Relevant electronic databases were consulted to identify appropriate studies using a set of search strings. In total, 12 out of 4971 publications were included in this review. The reported coating systems were assigned to several subgroups according to their characteristics: metallic, ceramic and composite. Notwithstanding the differences in study characteristics (animal model, implantation period, reported outcomes), it was noticed that several coatings improve the soft‐tissue integration as compared to pristine titanium. Porous titanium coatings having only limited pore sizes (<250 μm) do not support dermal fibroblast tissue attachment. Yet, larger pores (>700 μm) allow extensive vascularized soft‐tissue infiltration, thereby supporting cell attachment. Nanostructured ceramic coatings are found to reduce the inflammatory response in favor of the formation of cell adhesive structures, that is, hemidesmosomes. Biomolecule coatings seem of particular interest to stimulate the soft‐tissue behavior provided that a durable fixation to the implant surface can be ensured. In this respect, fibroblast growth factor‐2 entrapped in a biomimetic apatite coating instigates a close to natural soft‐tissue attachment with epidermal collagen fibers attaching almost perpendicular to the implant surface. However, several studies had limitations with respect to coating characterization and detailed soft‐tissue analysis, small sample size and short implantation periods. To date, robust and long‐term in vivo studies are still lacking. Further investigation is required before a clear consensus on the optimal coating system allowing enhancing the soft‐tissue seal around percutaneous titanium implants can be reached.

**Abbreviations**
Ag‐HA: silver substituted hydroxyapatite
BAHI: bone anchored hearing implant
cp: commercially pure
DLC: diamond like carbon
EMD: enamel matrix derivative
ECM: extracellular matrix
FGF‐2: fibroblast growth factor 2
Fn: fibronectin
HA: hydroxyapatite
OHAT: Office of Health Assessment and Translation
pTi: porous titanium
PDGF: platelet derived growth factor
PGA: poly(l‐glutamic) acid
PLL: poly(l‐lysine)
rhPDGF: recombinant human platelet derived growth factor
SEM: scanning electron microscopy
Si‐HA: silicon substituted hydroxyapatite
TEM: transmission electron microscopy
Ti: titanium
TiO_2_
: titania

## 1 INTRODUCTION

Titanium (Ti) implants are used in many different medical specialties, such as oro‐cranio‐maxillo‐facial surgery, dentistry, orthopedic surgery, and neurosurgery. They are used to replace bone tissue, stabilize bone segments or anchor prostheses in load‐bearing and non‐load‐bearing conditions. To guarantee implant stability, a secure and lifelong anchoring in the native surrounding bone, that is, osseointegration, is required. This is defined as the direct structural and functional connection between living bone and the surface of a load‐bearing implant without an intervening soft‐tissue layer.^1^ For applications in which the Ti‐implant is penetrating the skin to be connected to an extracorporeal part (e.g., in bone anchored hearing implants, dental implants, maxillofacial and orthopedic devices), not only osseointegration is needed, also a firm interface between implant and surrounding soft tissue is an important prerequisite for survival.^2^, ^3^, ^4^ The formation of a long‐standing biological barrier, with direct attachment of keratinized tissue to the implant surface, is necessary for long‐term implant success and viability.^2^, ^3^, ^4^, ^5^

Over the years, the concept of osseointegration of Ti implants in the host bone has already been extensively researched and described.^1^, ^6^, ^7^ The soft tissue integration of Ti, however, has been far less studied, although several authors have highlighted the importance of a firm soft‐tissue seal to optimize implant survival.^2^, ^7^, ^8^ Ideally, the epithelial‐implant interface is characterized by a thin soft‐tissue capsule including only a low number of inflammatory cells and fibroblasts. Furthermore, the collagen fiber orientation should be perpendicular or oblique to the implant surface^3^, ^9^, ^10^ otherwise, no direct soft tissue‐implant adherence is achieved.^11^

The implant surface plays a critical role in achieving a desirable cell and subsequent tissue response around implants. It is well accepted that the excellent biocompatibility of Ti and its alloys, owing to the presence of a protective oxide layer on the surface which remains highly stable even in the hostile biological environment, is at the basis of a direct bone apposition favoring osseointegration.^12^ Yet, achieving a permanent direct attachment of soft tissue seems more challenging.^8^ Animal experiments have revealed a barrier epithelium in direct contact with the TiO_2_ surface through hemidesmosomes. But collagen fiber bundles remain parallel to the implant surface and not perpendicular. Hereby a true chemical and therefore mechanical bonding to the titanium surface is not established.^13^

Surface modification of Ti to fine‐tune the surface physicochemical properties has been suggested to augment soft tissue integration.^5^ However, conventional surface modification techniques such as bead blasting, etching or anodization, alter the original surface of the substrate. Coatings do not have this effect. Rather, coating enables the complete coverage of the pristine metal surface with a biologically active material that encourages the host cell interaction, without modifying the original surface.^14^ Several coatings, mainly materials mimicking the components of living tissue, have been investigated for their potential to activate epithelial and/or fibroblast functions, such as inorganic CaP based coatings or biological coatings of extracellular matrix (ECM) components or growth factors.^5^ Yet, many of these studies only involve in vitro research, which sometimes varying outcomes. Moreover, the clinical significance of in vitro results is controversial because methodologies often do not consider the complexity of the in vivo situation.

With this systematic review focused on in vivo evaluation, we aim to gain more insight into the effect of coatings on the Ti implant‐keratinized tissue interface characteristics with the purpose of identifying those coatings that significantly improve the peri‐implant seal in vivo and therefore are most promising for further clinical investigation.

## 2 METHODS

### 2.1 Eligibility criteria

Studies eligible for this review were: original research papers, case reports, (non‐) randomized control trials and prospective and retrospective studies/case series, systematic reviews and meta‐analyses. Technical notes, editorials, letters to the editor, opinions or commentaries, which did not present original data were withheld. Only studies regarding the effect of coated Ti implants on the keratinized tissue seal were included. Studies solely researching the interface between Ti and bone were excluded. If studies reported results concerning the effect on soft tissue and osseointegration, only the keratinized results were accounted for. Only in vivo research was considered, this included animal studies as well as studies involving human subjects. In vitro research on soft tissue healing and fibrosis was not taken into account, as these studies often lack consensus. Furthermore, research methods applied for in vivo and in vitro studies differ too much, thereby hindering a reliable comparison of the results. No restrictions with respect to the publication date were imposed. Only the English, German, French and Dutch literature was checked.

### 2.2 Information sources and search strategy

**FIGURE 1:**
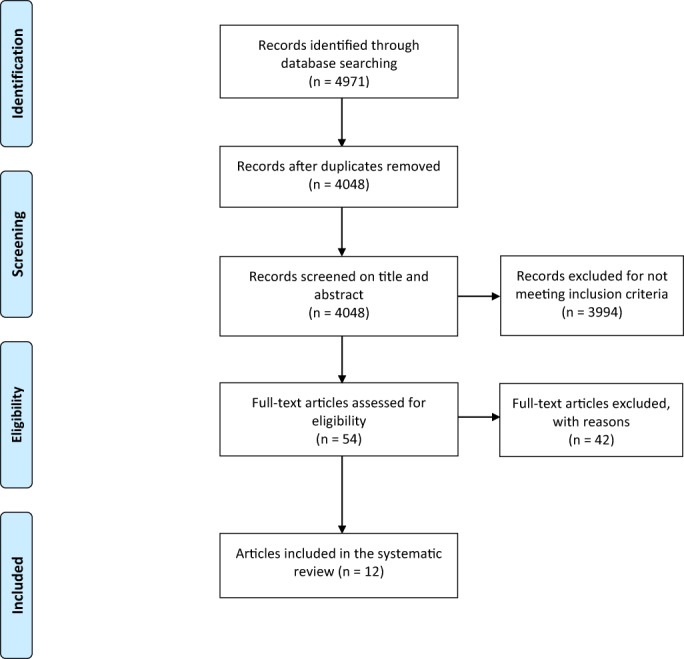
Flowchart with a detailed overview of the search strategy and study selection procedure

The systematic literature search was performed using the following electronic databases: PubMed Central (www.ncbi.nlm.nih.gov/pubmed), Cochrane Library (www.cochranelibrary.com), Embase (www.embase.com), and Web of Science (https://www.webofknowledge.com). Next, the following trial registers were screened to include the most accurate and up‐to‐date research: clinicaltrials.gov and trialregister.nl. Finally, to extend the search, reference lists of the relevant studies were screened for relevant articles and filtering cited articles. A detailed overview of the Boolean searches together with the search results is given in Table 1 and Figure 1.

**TABLE 1:** Overview of the detailed Boolean search, that is, a database‐appropriate syntax in combination with the selected search terms, and the search result for the consulted databases

Database	Boolean search	Search result
PubMed Central	(((((((((((((((((((((((((((((titanium[MeSH Terms]) AND coating) OR organic) OR ameloblastin) OR laminin) OR glycosaminoglycans[MeSH Terms]) OR extracellular matrix proteins[MeSH Terms]) OR growth factors[MeSH Terms]) OR DNA) OR biphosphonate) OR antibiotics[MeSH Terms]) OR antimicrobial agent[MeSH Terms]) OR biopolymer[MeSH Terms]) OR inorganic) OR calcium phosphate) OR titanium dioxide) OR nitride) OR metals[MeSH Terms]) OR carbon) OR bioactive glass) OR bioactive ceramics) OR diamond) OR silk) OR bioceramics) OR silica) OR methicone) OR triethoxysilane) AND keratinized tissue)) NOT osseointegration	4235
Embase	(titanium AND coating AND keratinized OR keratinised) NOT ('osseointegration'/exp OR osseointegration)	270
Cochrane Library	"titanium" in All Text AND "coating" in All Text AND ("keratinised" in All Text OR keratinized in All Text) NOT "osseointegration" in All Text	52
Web of Science	ALL FIELDS: (titanium) AND ALL FIELDS: (coating) AND ALL FIELDS: (keratinized) NOT ALL FIELDS: (osseointegration)	290
www.clinicaltrials.gov	Titanium AND coating	93
www.trialregister.nl	Titanium	31

### 2.3 Study records

The selection process (screening, eligibility, and data extraction) was carried out by two independent researchers (C. V. D. B. and B. Z.). Articles were included through title and abstract screening. If eligible, the full text was analyzed and assessed for inclusion. All articles eligible for the systematic review were stored electronically in a full‐text version.

### 2.4 Risk of bias in individual studies

An assessment of internal validity, performance, selection, and other types of bias for individual human and animal studies was performed using the OHAT Risk of Bias Rating Tool for Human and Animal Studies. The analysis was done at study level and was carried out by two independent reviewers (C. V. D. B. and B. Z.).

## 3 RESULTS

### 3.1 Study selection

The search yielded a total of 4971 articles, a detailed overview of the search results per database is given in Table 1. After initial screening of title and abstract, 54 records were found. The full‐text articles were further assessed for eligibility and a total of 12 studies could be included in this systematic review. The overall quality of the studies under review was assessed using the OHAT Risk of Bias Rating Tool for Human and Animal Studies. Results deviated but were acceptable as given in Table 2.

**TABLE 2:** To assess the risk of bias in the included studies, the OHAT risk of bias framework was used

Author (year)	Selection bias	Confounding bias	Performance bias	Attrition bias	Detection bias	Selective reporting bias
Pendegrass (2006)		+	−	+	++	+
Glauser (2006)		+	−	+	+	+
Welander (2007)	−		−	+	+	+
Rossi (2008)		++	−	+	+	+
Werner (2009)	+		−	+	+	+
Mutsuzaki (2012)	−	+	+	+	++	+
Bates (2013)	−	−	−	+	+	−
De Wilde (2013)			−	+	+	+
Larsson (2015)	−		−	−	+	+
Høgsbro (2017)	−		−	+	−	+
Chimutengwende‐Gordon (2017)	−	+	+	+	+	+
Li (2020)	+		+	+	+	+

*Note*: “++”: definitely low risk of bias; “+”: probably low risk of bias; “–”: probably high risk of bias; “– –”: definitely high risk of bias.

### 3.2 Study material

A detailed evaluation and data extraction was performed for the 12 selected studies, the major characteristics are given in Table 3. The study design varied greatly in terms of animal model and time of exposure. Of the selected studies, three involved humans and nine were animal studies, distributed as follows: rabbits (1), dogs (3), rats (1), sheep (2), goats (1), and mice (1). Exposure time varied between 7 days and 12 months. While the Ti material used for the implant was mostly commercially pure (cp) Ti or a Ti6Al4V alloy, the implant geometry differed between studies going from cylinders or screws to discs. Various coating materials were used on the Ti implants: mainly ceramics (hydroxyapatite (HA), titania (TiO_2_), diamond‐like carbon (DLC)) or metals (porous Ti [pTi]), but also biomolecules (growth factors) as well as composites (combining organic and/or inorganic materials). Depending on the applied coating technique, the thickness of the coatings varied extensively, ranging from 20 nm to 100 μm. For a comprehensive evaluation, results were compiled into specific subgroups based on the coating material: metallic coatings, ceramic coatings, and composite coatings.

**TABLE 3:** This table gives more information about the included studies and their major characteristics

Author (year)	Animal model	Implant geometry	Titanium grade	Coating type	Coating thickness	Coating technique	Time of exposure
Pendegrass (2006)	24 Sarneen goats	Screw (∅ > 4.2 mm × 40 mm) without or with flange (∅ 10 mm)	Ti6Al4V	Screw: HA pTi pTi + HA pTi + DLC pTi + HA + DLC Flange: HA	HA: 70 μm pTi: 70–100 μm DLC: 2–4 μm	HA or pTi: plasma spraying DLC: chemical vapor deposition	4 weeks
Glauser (2006)	5 humans	Screw (M 2.3 mm × 10 mm)	cp Ti	TiO_2_ (microporous)	—	Microarc oxidation	8 weeks
Welander (2007)	6 Labrador dogs	Screw (M 3.75 mm × 12.8 mm)	cp Ti	Collagen type I (purified porcine) in TiO_2_	40 nm	Entrapment in anodic oxide layer by: 1. anodization in collagen solution 2. immersion in a collagen solution	4 weeks 8 weeks
Rossi (2008)	6 Beagle dogs	Screw (M 4.1 mm × 8 mm)	cp Ti	TiO_2_	380 nm (for five layers)	Sol–gel dip‐coating (ITI implant)	8 weeks
Werner (2009)	9 Beagle dogs	Screw (M 3.3 mm × 6 mm)	cp Ti	pTi Adhesion peptide (Laminin‐5 derived peptide) in PLL/PGA polyelectrolyte film	pTi: ca. 400–600 μm PLL/PGA/laminin‐5: 80 nm	pTi: sintering by condensed electrical discharge Laminin‐5 derived peptide: immobilization by chemical grafting	6 months
Mutsuzaki (2012)	16 Japanese White rabbits	Screw (M 4.0 mm × 30 mm)	cp Ti	FGF‐2 − apatite composite layers	0.8–1.7 μm	Entrapment in apatite by biomimetic deposition	4 weeks
Bates (2013)	12 Dark Agouti rats	Screw (M 3.3 mm × 10 mm)	cp Ti (grade 4)	TiO2 rhPDGF or EMD	—	Microarc oxidation Adsorption by immersion	4 weeks 8 weeks
De Wilde (2013)	13 Humans	Screw (M 1.5 mm × 8 mm)	cp Ti	HA	20 nm	Dip‐coating followed by heat treatment	8 weeks
Larsson (2015)	8 sheep	BAHI implant system	cp Ti	HA	80 μm	Plasma spraying	4 weeks
Høgsbro (2017)	25 Humans	BAHI implant system	cp Ti	HA	80 μm	Plasma spraying	1 week up to 12 months
Chimutengwende‐Gordon (2017)	6 Sheep	Screw (M 4.2 mm × 45 or 47 mm) with flange (∅ 11 mm × 4.3 or 1 mm)	Ti alloy	pTi pTi + HA pTi + HA/Fn pTi + Ag‐HA pTi + Ag‐HA/Fn	pTi: ‐ HA: 12–76 μm Ag‐HA: 24–100 μm Fn: ‐	pTi: laser‐sintering HA or Ag‐HA: electrolytic deposition Fn: adsorption by immersion	4 weeks
Li (2020)	Mice (amount not specified)	Cylinder (Ø 2 mm × 15 mm)	cp Ti	HA Si‐HA	3 μm	Alkali‐heat treatment followed by hydrothermal treatment	4 weeks

*Note*: If no information was given concerning the characteristics a “–” was placed.

Abbreviations: Ag‐HA, silver substituted hydroxyapatite; BAHI, bone anchored hearing implant; C, carbon; cp, commercially pure; cp, commercially pure; DLC, diamond like carbon; EMD, enamel matrix derivative; FGF‐2, fibroblast growth factor 2; Fn, fibronectin; HA, hydroxyapatite; PGA, poly(l‐glutamic) acid; PLL, poly(l‐lysine); pTi, porous titanium; rhPDGF, recombinant human platelet derived growth factor; Si‐HA, silicon substituted hydroxyapatite; Ti, titanium.

#### 3.2.1 Subgroup 1 – Metallic coatings

The formation of a firm interface between a Ti implant surface and the surrounding soft tissue is vital for a long‐term implant survival. However, in the absence of a firm soft‐tissue seal, epithelial downgrowth can destabilize the interface. The formation of a non‐adherent, fibrous tissue layer can occur, which decreases implant survival rates. Implant failures have been reduced by various implant designs and by employing different thin, porous metallic coatings on the bare metal surface. Especially, pTi has been investigated to promote soft tissue ingrowth.

pTi has been described by several papers, mainly to investigate the effect on epithelial/subepithelial downgrowth, but outcomes varied. For example, Pendegrass et al. modified machined Ti6Al4V pins with a plasma sprayed pTi layer (thickness: 70–100 μm; pore size: 30–250 μm), but neither the epithelial downgrowth nor the percentage of subepithelial layer attachment was influenced. On the contrary, Ti particle debris detached from the pTi coating and was observed in the soft tissue surrounded by lymphocytes which could indicate chronic inflammation (depending on the number of lymphocytes present). With respect to soft‐tissue ingrowth, only sporadic areas of close attachment and ingrowth were observed in the sub‐epithelium. Alternatively, Werner et al. modified the smooth cp Ti surface at the transmucosal part of ITI implants (Straumann AG, Basel, Switzerland) with a pTi coating obtained by sintering of cp Ti beads using electrical discharges (thickness: ca. 400–600 μm; bead size: 125–160 μm).^15^ A good soft tissue healing without any sign of inflammation was confirmed and implants were well integrated with the surrounding tissues (bone, connective tissue and epithelium) with cells able to colonize the microporosities of the pTi coating. Similar results were found by Chimutengwende‐Gordon et al., who compared transcutaneous pins with laser‐sintered pTi (pore size: 700 μm) flanges to pins with drilled Ti flanges.^16^ It was found that the pTi coating reduced epithelial downgrowth, but the epithelial attachment was similar for both flange materials. Yet, an increased dermal attachment could be observed for pTi flanges and the median percentage soft tissue fill and median density of fibroblast nuclei within the inner pores of the implant was significantly increased for pTi coated as compared to drilled flanges.

#### 3.2.2 Subgroup 2 – Ceramic coatings

Ti and its alloys meet many of the biomechanical requirements for load‐bearing implants. Moreover, the stable oxide layer that forms at the surface minimizes metal ion release into the biological environment, which largely explains its biocompatibility. However, the material remains bioinert and therefore does not actively support soft tissue adhesion. Owing to their excellent bioactivity, many research efforts have been devoted to ceramic coatings for various applications in soft tissue regeneration.

An often considered ceramic coating material is HA obtained by a variety of processing routes. Pendegrass et al. investigated the effect of plasma sprayed HA coatings (thickness: 70 μm; average roughness *R*_a_ = 2.4 μm with *R*_a_ being the arithmetic average of the absolute values of the profile heights) on the soft‐tissue interface around bone anchored transcutaneous Ti6Al4V implants in goats. HA coatings did not seem to significantly reduce epithelial downgrowth or improve epithelial or subepithelial attachment when compared to pristine implant surfaces, yet the authors attributed this to inaccurate positioning of the HA coatings within the soft tissues. This does not correlate to the results obtained by Larsson et al. who investigated the effect of a plasma sprayed HA coating (thickness: 80 μm) on smooth cp Ti bone anchored hearing implant (BAHI) abutments fixed to Ti implants using a sheep model.^17^ Here, it was found that after 4 weeks, epidermal downgrowth and pocket depths were significantly reduced for HA coated abutments, hereby demonstrating improvements in soft‐tissue integration regarding the intimate dermal junction. However, in a clinical study including 25 human subjects, Høgsbro et al. evaluated the keratinized tissue‐implant interface for plasma sprayed HA coatings (thickness: 80 μm; *R*_a_ = 7 μm) on smooth cp Ti BAHI abutments.^18^ After a follow‐up period of 1 year, it was concluded that the HA coating did not significantly improve the soft‐tissue reaction in comparison to smooth Ti abutments. Alternatively, to the relatively rough plasma sprayed coatings, implants featured with a nanostructured HA coating have been investigated as well. A study in humans by De Wilde et al. investigated the soft‐tissue response to nano‐HA (thickness: 20–30 nm; average roughness *S*_a_ = 1 μm with *S*_a_ being the arithmetic mean of the absolute values of the surface departures from the mean plane) coated cp Ti dental implants installed in the jawbone.^19^ After 8 weeks of implantation, implants were removed and immunologically and histologically evaluated. No significant differences in terms of inflammatory response in the transmucosal regions were found for the nano‐HA coated surfaces as compared to uncoated Ti. Different results were observed for nanostructured HA coatings applied by a combination of alkali treatment and subsequent hydrothermal treatment. Li et al. investigated the effect of nanorod HA coatings (thickness: 3 μm; *R*_a_ = 0.24 μm) on cp Ti on skin integration for percutaneous rods in a mice model.^20^ Whereas a thick fibrous capsule of 400 μm was observed at the soft tissue – implant interface around uncoated cp Ti rods, the capsule thickness decreased to about 100 μm for nanorod HA coated implants. This effect was further improved when silicon was substituted into the nanorod HA coatings (Si‐HA), as illustrated by an even more reduced epithelial downgrowth and the absence of a fibrous capsule around the implant, indicating a tighter seal between the surface and the underlying dermis.

TiO_2_ is another ceramic that is often investigated as coating material to improve the soft‐tissue response to percutaneous implants. For example, Glauser et al. prepared TiO_2_ coatings on cp Ti by means of microarc oxidation which resulted in a characteristic microporous oxidized surface layer.^21^ These dental implants were installed in five human patients and compared to a machined or acid‐etched surface following a transmucosal healing period of 8 weeks. Implants were harvested with a layer of surrounding hard and soft tissue and the histomorphometric characteristics of the peri‐implant soft‐tissue barrier were investigated. It was observed that TiO_2_ modified implants reduced downgrowth of epithelia as compared to machined Ti implants, yet the connective tissue was oriented circumferentially to the implant surface without any perpendicularly oriented collagen fibers directly contacting the implant surface. Bates et al. also prepared TiO_2_ coatings by means of microarc oxidation.^22^ Six implants were installed in a rat model and harvested after 4 and 8 weeks. At both time points, histological assessment showed connective tissue in intimate contact with the implant surface. After 8 weeks, a greater depth of penetration into the implant grooves was seen when compared to 4 weeks. However, no perpendicular collagen fibers were seen. A layer of adipose tissue was noted adjacent to the fibrous tissue.

Alternatively, also sol–gel derived TiO_2_ coatings have been considered to improve the peri‐implant tissue response. As such, Rossi et al. evaluated nanoporous TiO_2_ thin films (thickness: 380 nm; *S*_a_ = 0.26 μm) coated on the smooth cp Ti surface at the transmucosal part of ITI implants (Straumann AG, Basel, Switzerland) in a beagle dog model.^23^ Scanning electron microscopy (SEM) evaluation after 8 weeks of implantation showed numerous gingival cells attached to the coated implant surface. In all specimens, keratinized oral epithelium was seen that was continuous with the junctional epithelia facing the implant surface. Furthermore, histological examination showed a mild or absent inflammatory reaction in peri‐implant connective tissues around the surface coated implants. In contrast, unmodified surfaces were seen to instigate a capsule‐like structure leading to minor cell adhesion as illustrated by a total detachment of the junctional epithelium from the implant surface in 45% of the reported implants. When analyzed by transmission electron microscopy, dense plaques of hemidesmosomes were revealed facing the surface‐treated implants.

Finally, DLC has been suggested as a coating material exhibiting low surface energy and concomitantly low bacterial adhesion, originally only for external parts of transcutaneous implants not in contact with the soft tissues in order to reduce infections. Pendegrass et al. compared DLC‐coated sandblasted or grooved Ti6Al4V to uncoated machined Ti6Al4V bone‐anchored transcutaneous implants in a goat model for 4 weeks. There were no clinical signs of infection, but DLC coatings did not seem to affect the epithelial downgrowth or epithelial/subepithelial layer attachment.

#### 3.2.3 Subgroup 3 – Composite coatings

To further tailor the coating properties to the specific requirements of a targeted application, combinations of two or more materials that form a layered or mixed structure have been proposed. These so‐called composite coatings synergistically combine the functionalities of both materials for an improved therapeutic effect which can otherwise not be realized by a traditional coating. Based on the nature of the coating materials, inorganic–inorganic, organic–inorganic and organic–inorganic composite coatings have been identified in the here reviewed in vivo studies.

### 3.3 Inorganic–inorganic coatings

One coating type involving multiple inorganic materials that was investigated in several papers was the layered combination of pTi, for an improved ingrowth of soft‐tissue in the porous layer, with a HA, to improve fibroblast attachment (Pendegrass et al). In an in vivo study in goat tibiae, Pendegrass et al. did not observe differences in soft‐tissue morphology around transcutaneous machined Ti6Al4V implants whether or not coated with a plasma sprayed pTi layer (thickness: 70–100 μm; pore size: 30–250 μm) including a plasma sprayed HA topcoating (thickness: 70 μm). Downgrowth and epithelial or subepithelial tissue attachment was not significantly different. Here as well, a DLC coating was considered for the external parts, yet a decreased epithelial and subepithelial layer attachment was observed, however, this was not statistically significant. Alternatively, Chimutengwende‐Gordon et al. investigated transcutaneous pins with laser‐sintered pTi (pore size: 700 μm) flanges with an electrochemically deposited HA topcoating (thickness: 30–76 μm) in an in vivo sheep model. Other than the plasma spraying method, which is a line‐of‐sight process, electrochemical deposition allows to also coat the inner pores of the pTi (thickness: 12–55 μm). Moreover, it is a versatile process enabling incorporation of substituting ions, such as silver, within the HA (Ag‐HA) for antimicrobial activity upon release. Whereas pristine laser‐sintered pTi flanges already showed an improvement in comparison with drilled flanges including a plasma sprayed HA topcoating (see subgroup 1), inclusion of HA and Ag‐HA topcoatings on pTi flanges did not further reduce epithelial downgrowth nor was the epithelial or dermal attachment and soft‐tissue fill or fibroblast nuclei density in the inner pores further improved.

### 3.4 Organic–inorganic coatings

Organic biomolecule coatings have been of keen interest to improve the implant‐soft tissue seal by targeting enhanced adhesion and proliferation of epithelial cells and fibroblasts.^5^ Yet, in literature, different methods are used to apply these biomolecules on the implant surface. Methods used to attach biomolecules to the implant surface can typically be classified into three categories, that is, physical adsorption to the substrate, physical entrapment in an additional coating layer or chemical grafting to the implant substrate through irreversible chemical links.^24^ All these categories were also represented in the here reviewed in vivo studies.

Firstly, Chimutengwende‐Gordon et al. applied fibronectin (Fn), an anchoring protein regulating cell attachment and mobility, through physical adsorption by means of simple immersion on HA or Ag‐HA coated pTi flanges (laser‐sintered pTi flanges, pore size: 700 μm; electrochemically deposited HA topcoating, thickness: 30–76 μm, see above) on transcutaneous pins. These Fn modified HA (HA/Fn and Ag‐HA/Fn) surfaces were evaluated in a sheep model for 4 weeks. Epithelial downgrowth and attachment was similar as observed for pTi, pTi + HA and pTi + Ag‐HA as well as the soft‐tissue fill and density of fibroblast nuclei within the inner pores of the implant. Dermal attachment to Fn modified surfaces, however, was improved in comparison to their unmodified counterparts.

Alternatively, to this simple adsorption method, Welander et al. partially integrated the structural ECM protein collagen type I in an anodically formed oxide layer on a cp Ti implant surface by an electrochemical process.^25^ The soft‐tissue reaction was evaluated in a beagle dog model for 4 and 8 weeks. It was found that the vertical dimensions of the epithelial and connective tissue components of the soft tissue/implant interface were similar for collagen‐coated implants as compared to cp Ti controls. The epithelial cell attachment was also similar for both conditions. As SEM analysis could not identify the collagen coating anymore after 4 weeks, the authors hypothesize that the coating degraded prematurely. Mutsuzaki et al. used a similar approach to incorporate fibroblast growth factor‐2 (FGF‐2), known to facilitate fibroblast proliferation and angiogenesis which promotes cell interaction, viability and attachment, in a calcium phosphate coating by means of biomimetic deposition.^26^ In vitro testing confirmed that FGF‐2 was released from the coatings for at least 4 days, while retaining its bioactivity. The in vivo effect of FGF‐2/apatite composite coatings on soft‐tissue healing around percutaneous cp Ti screws was evaluated in rabbits. The FGF‐2/apatite composite coating seemed to have a beneficial effect on the soft‐tissue/implant interface. An interfacial tissue layer of 100 μm in thickness was formed consisting of an inner and outer layer. While the inner cell layer was directly attached to the FGF‐2/apatite composite coating and consisted of thin and stretched cells (0.8–1.7 μm thick and 16–33 μm long), the outer layer consisted of Sharpey fiber‐like tissue with many blood vessels and collagen fibers inclined at angles from 30 to 40° to the screw surface.

Bates et al. investigated the effect of platelet derived growth factor (PDGF) and enamel matrix derivative (EMD) coatings on the connective tissue attachment to TiUnite (TiO2 coating) implants (Nobel Biocare AB, Göteborg, Sweden) in a rat model. Coatings were applied by physical adsorption by simply immersing the implants in the growth factor solution for 30 s immediately prior to implantation. After 4 weeks of implantation, the connective tissue infiltration around rhPDGF‐coated implants was significantly increased in comparison to control and EMD‐coated implants. However, after 8 weeks, this difference was no longer significant. Histological assessment showed the presence of an adipose‐like layer at the implant surface. No perpendicular attachment of collagen fibers or attachment of fibroblast directly to the implant surface was seen using histological assessment.

### 3.5 Organic–organic coatings

Werner et al. incorporated a laminin‐5 derived peptide at the pTi modified abutment surface of ITI implants (Straumann AG, Basel, Switzerland) by means of chemical grafting on an intermediate multi‐layered poly(l‐lysine) (PLL) / poly(l‐glutamic) acid (PGA) polyelectrolyte film (PLL/PGA coating thickness: 60 nm; overall thickness: 80 nm). Laminin‐5 is involved in the nucleation and maintenance of hemidesmosomes, the adhesion structures that bind epithelial to the implant surface and are thus involved in the structural scaffolding of epithelial tissues. The authors hypothesized that a peptide including the amino acid sequence representing the integrin‐dependent cell‐adhesion site on laminin‐5 could further improve the cell‐adhesion properties of the experimental pTi coating (sintering by electrical discharging, thickness: ca. 400–600 μm; bead size: 125–160 μm; see subgroup 1). Laminin‐5‐functionalized abutment surfaces were evaluated in vivo in a dog model for 6 months and compared with pristine pTi surfaces. Efficient colonization of epithelial cells into the microporosities of the pTi surfaces was observed in both cases. Yet, the study did not include a histological analysis of the soft‐tissue organization and remains inconclusive from this perspective.

## 4 DISCUSSION

Soft‐tissue adhesion at the skin/implant interface is crucial for the long‐term success of percutaneous implant treatments. However, the formation of a firm bioseal around Ti implants is not easily achieved. Host reactions following implantation of foreign biomaterials lead to acute and chronic inflammation resulting in the formation of granulation tissue. This granulation tissue is separated from the implant by a one‐ to two‐cell layer of monocytes, macrophages and foreign body giant cells.^27^ This layer matures and a fibrous capsule formation is seen as the end‐stage healing response.^27^ This capsule inhibits a direct adherence of neighboring soft tissues to the implant surface, which is fundamental to establish a protective barrier against the external environment and harmful microbial invasion and, thus, infection.^28^

Epithelial downgrowth is a major factor that destabilizes the soft tissue‐implant interface. Downgrowth occurs as a direct result of the so called “free edge effect”.^29^ Due to the absence of neighboring cells, no signals for proliferation and migration occur and epithelial cells favor to establish layer continuity with the wound edge epithelial cells. Ideally, the surface of epithelium‐penetrating implants should impede this apical epithelial migration to ensure implant success. According to Winter et al. the key lies in the dermis.^29^ If the dermal cells were to quickly attach to the implant surface, the epithelial cells would not migrate between the dermis and the implant surface. This is exactly what can be observed in percutaneous interfaces around teeth or deer antlers, which can be considered the natural analogues of percutaneous implants. However, one of the major differences compared to Ti implants is the mechanical attachment of the inserting collagen fibers, also called Sharpey's fibers. Rather than engulfing the tooth or antler, these Sharpey's fibers insert perpendicular to the surface of the tooth and provide a firm connective tissue connection. These same angles of implantation must occur to provide the same bioseal on implant level and to prevent apical epithelial recession/downgrowth.^4^, ^8^, ^26^

Host reactions and healing response are governed by the cellular response to the protein layer formed at the implant surface upon contact with the body fluids. As the composition and conformation of this protein film is determined by the physicochemical surface properties, surface modification of the transcutaneous region of an implant is an intensively researched strategy to improve the soft‐tissue integration.^2^ Covering the pristine implant surface with biologically active coatings is a flexible approach allowing introducing the necessary cues to limit foreign body reactions in favor of soft‐tissue cell attachment. Many different coatings have been engineered and investigated over the years, but mainly in vitro research was conducted.^5^, ^8^ This systematic review identified only 12 in vivo studies addressing coatings for soft‐tissue integration around percutenanous Ti implants. Nevertheless, these covered a wide range of coating strategies, either addressing the surface topography (pTi) or chemistry (ceramics) or introducing truly biologically active organic components (biomolecules) at the implant surface, as well as combined approaches.

Porous structured Ti surfaces are considered for soft‐tissue integration as these offer an enlarged specific surface area available for cell attachment and tissue ingrowth.^8^ Overall, the here reviewed pTi coatings showed a good soft‐tissue reaction, although care should be taken to avoid Ti particle release from the coatings, a complication commonly associated with plasma sprayed Ti coatings and which was found to trigger inflammation.^4^, ^30^ The effective establishment of a firm seal at the implant‐keratinized tissue interface varied between studies. pTi coatings did not seem to improve soft‐tissue integration for straight implants, but a reduced epithelial downgrowth, increased dermal attachment and ingrowth of vascularized soft tissue into the pores was reported for flanged implants with pTi structured flanges.^4^, ^31^ Although the implant design was different and a meshed implant collar was previously shown to reduce epithelial downgrowth, differences might also be explained by a discrepancy in pore size in the pTi structures used.^32^ It has been previously hypothesized that the anatomical and physiological characteristics associated with soft tissues require a more open pore structure in order to maintain viable tissue as compared to bone, where 100 μm pore size are considered the lower limit for osseointegration.^30^ Indeed, whereas almost no dermal fibroblastic tissue attachment to the implant surface was seen for pore sizes between 30–250 μm, extensive tissue infiltration was observed when the pore size increased to 700 μm.^4^, ^16^ This is in correspondence with previous findings by LaBerge et al. who observed fibrous encapsulation for porous coated CoCr implants having pore diameters of 300 μm, while pore diameters of 900 μm became infiltrated with a vascularized soft tissue.^33^ This was also confirmed by another study by Chimutengwende‐Gordon et al., where it was found that porous Ti implants having pore sizes of 250 μm did not allow soft‐tissue infiltration. It can thus be assumed that the low pore interconnectivity and smaller pores associated with plasma sprayed pTi coatings was not sufficiently promoting cellular infiltration and vascular ingrowth, whereas the fully interconnected open porous structures obtained by laser‐sintering, a metal 3D printing technology, facilitated the invasion of blood vessels into the structure which supported early attachment of cells.^34^

Altering the surface chemistry of an implant may be considered more effective in controlling cellular behavior than surface topography and is therefore another valuable approach to fine‐tune soft‐tissue integration.^35^ Inspired by osseointegrative strategies, a frequently considered coating material was HA. Given its close resemblance with the inorganic phase in bone, applications of HA have been mostly related to hard tissue repair. However, several studies have demonstrated its ability to firmly integrate with dermal tissues.^9^, ^36^ Yet, when applied as coating, HA seemed to perform poorly in vivo with respect to soft‐tissue response. Earlier studies, which mainly involved plasma sprayed HA, did not observe a direct soft‐tissue contact with the implant surface. However, it should be noted that plasma sprayed HA coatings exhibit a relatively rough surface (*R*_a_ = 2–7 μm). Alternatively, wet‐chemical methods allow introducing a nanotopography as also found in natural tissues. Hydrothermally grown nanorod HA coatings (*R*_a_ = 0.24 μm) significantly reduced the inflammatory reaction to pristine Ti, especially when the surface chemistry was further modified by substituting silicon in the HA which accelerated the biosealing.^20^

A similar beneficial effect of nanotopography was also observed for TiO_2_ coatings. Both microarc oxidized and nanoporous sol–gel derived TiO_2_ coatings reduced epithelial downgrowth as compared to pristine Ti.^21^, ^23^ But, whereas for microarc oxidized TiO_2_ coatings collagen fibers were circumferentially oriented to the implant surface without direct contact, nanoporous sol–gel derived coatings instigated an immediate contact of connective tissue as revealed by the presence of hemidesmosomes. The exact mechanism of soft‐tissue attachment, however, remains unclear. It has been shown that the Ti‐OH functional groups present on the anatase and rutile structured titania gel render it a high‐energy surface. These TiOH‐groups initiate calcium phosphate nucleation, which in turn facilitates adsorption of proteins, for example, fibronectin which mediates the adhesion and spreading of connective tissue cells for a good soft‐tissue integration. Furthermore, the high surface energy presented by these coatings also limit capsule formation which results in a close contact or even direct attachment of soft tissue to the implant surface.^23^

Alternatively, instead of fine‐tuning the surface topography and chemistry in order to modulate protein adsorption from the body fluids upon implantation, recent strategies aim to already biofunctionalize the implant surface prior to implantation using bioactive proteins that trigger known cellular responses.^24^ A common approach is to include biomolecules at the implant surface that mimic the natural environment of these target cells (cell homing). As such, the adhesive structures through which cells adhere to the implant surface, that is, hemidesmosomes and internal basal lamina, have inspired the use of adhesion related proteins or their functional peptides, such as laminins and fibronectin, but also collagens as structural proteins within the ECM to which cells adhere have been considered.^5^ On the other hand, cell‐signaling molecules capable of mediating cell behavior crucial for tissue healing and regeneration, such as growth factors, represent another approach to obtain soft‐tissue integration. However, the way these biomolecules are presented to the surrounding media determines the success of such biomolecule coatings.^24^ Therefore, coating techniques should not alter the conformation or functionality of the biomolecules. On the other hand, good adhesion of the biomolecules to the implant substrate is also of paramount importance. Biomolecules mimicking the adhesive structures of the ECM are thought to enable a true mechanical attachment of collagen fibers but should in turn also firmly attach to the implant surface in order to guarantee a good soft‐tissue seal. But also, the effect of signaling molecules, which rely on their release from the surface to act on the target cells, can be enhanced if the molecules are retained at the surface over a longer period of time.

Simple physical adsorption of biomolecules to the implant surface is thought not to affect the biomolecule structure (and hence functionality) much, however, such coatings are only attached to the implant surface through weak interactions (van der Waals forces or electrostatic interactions) and are expected to solubilize upon contact with the body fluids during the early stages of implantation.^24^ This can explain why physically adsorbed coating of the anchoring protein Fn onto did not seem to improve soft‐tissue attachment to HA‐coated pTi transcutaneous pins.^16^ Similarly, rhPDGF coatings on TiUnite implants only showed a beneficial effect on the short‐term, as soluble growth factors are prone to rapid enzymatic degradation.^22^ For a more durable attachment and activity of the biomolecules, physical entrapment in an inorganic surface coating can be considered.^24^ However, the structural ECM protein collagen type I physically entrapped in an anodically grown oxide coating on cp Ti did not improve the soft‐tissue integration.^25^ The authors hypothesized that the collagen degraded prematurely. Promising results, on the other hand, were obtained for FGF‐2 which was physically entrapped in biomimetically deposited calcium phosphate coatings.^26^ Whereas in vitro release testing showed the controlled delivery of FGF‐2 for a prolonged period, the in vivo experiments confirmed its beneficial effect on soft‐tissue attachment. Authors reported Sharpey's fiber‐like structures running at 30–40° degrees inclination to the implant surface. However, it was not discussed how the ends of the collagen fibers inserted in the FGF‐2‐apatite composite layer. Alternatively, for a long‐lasting fixation of the biomolecules to the surface, covalent immobilization through irreversible chemical bonds can be applied.^24^ Laminin‐5 derived peptide coatings chemically grafted on pTi through an intermediate polyelectrolyte coating as described by Werner et al. showed an intimate implant‐soft tissue connection. However, the limited histological analysis performed in this preliminary study did not allow to distinguish differences with the soft‐tissue response to pristine pTi.

After considering the results of all reviewed studies, a clear conclusion on the optimal coating system to improve the soft‐tissue interface around percutaneous Ti implants and what this would indicate for human studies, cannot be drawn. Several factors hinder a straightforward comparison of the here presented studies. A first restriction is the limited surface characterization performed and inconsistency in reported coating properties. In fact, few papers went into detail about their surface characteristics. A reference was mostly made to earlier publications performed by the same research group, however this data was mostly limited to coating thickness and average roughness. We have recently made recommendations for a comprehensive surface characterization to correlate with the soft‐tissue response.^37^ Functional coatings tend to be fragile as compared to the high insertion forces applied during implantation and can lose activity upon sterilization or storage.

Another important restriction is that the methodologies for evaluating the soft‐tissue/implant interface varied significantly between studies, both in approach, profundity and histomorphometric analyses. As indicated above, dermal attachment is a prerequisite to prevent epithelial downgrowth. Unfortunately, not all studies compared the dermal and epidermal connection to coated and pristine implants individually. A comparison of the effect of the different coatings on the soft tissue interface was therefore difficult. Moreover, only a few studies incorporated detailed high‐resolution imaging allowing to analyze the orientation of dermal collagen fibers in contact with the implant surface, even though it is believed that a perpendicular insertion of fibers confirms the establishment of a firm bioseal. Another limitation that needs to be addressed is that most studies only report on a small sample size with a variable time of exposure. This could be due to various reasons, amongst others ethical considerations regarding use of animals in in vivo experimentation. A small sample size may reduce the statistical power of a study. In very small studies, there exists a possibility of (selection) bias. Therefore, the study quality was assessed by the OHAT risk of bias framework to evaluate risk of bias on study level in human and non‐human animal studies. All studies were acceptable according to this assessment. No studies were excluded solely based on sample size, as this can lead to loss of important data.

## 5 CONCLUSION

Overall, 12 publications reporting on the effect of implant coatings on the keratinized soft‐tissue interactions have been included in this systematic review. These studies varied significantly in terms of animal model, healing period, and measured outcomes. In addition, often only limited coating characteristics have been reported. Although a direct comparison was not possible, several valuable observations could be highlighted. When compared to the pristine Ti implant surface, metallic pTi coatings improve soft‐tissue integration depending on pore interconnectivity and especially pore size. Pore sizes >250 μm are required to enable dermal fibroblastic tissue attachment, while even larger pore sizes (>700 μm) enable infiltration of vascularized soft tissue and further support the attachment fiber rich connective tissue to the implant surface. For ceramic coatings, nanotopography, as opposed to a high surface roughness, appeared to be a key feature and was found to reduce inflammation, thereby positively triggering the formation of hemidesmosmes. Furthermore, several results indicated that for biomolecule coatings, a prolonged stable presentation of the biomolecule at the implant surface is required to allow a significant biological activity. Growth factors (FGF‐2) entrapped in biomimetically grown apatite induced the attachment of collagen fiber‐like structures running at 30–40° degrees inclination to the implant surface, a near perfect implant‐soft tissue interface. Besides some promising results, none of the included studies were able to indicate the formation an implant‐soft tissue seal with the same complexity as seen in nature. Therefore, further long‐term in vivo research is recommended, which should focus on a more comprehensive surface characterization, detailed in‐depth soft tissue analyses and their correlation.

## CONFLICT OF INTEREST

The authors declare no conflicts of interest.

## Data Availability

Data sharing is not applicable to this article as no new data were created or analyzed in this study.
